# The Global Action for Measurement of Adolescent health (GAMA) Initiative—Rethinking Adolescent Metrics

**DOI:** 10.1016/j.jadohealth.2019.03.008

**Published:** 2019-06

**Authors:** Regina Guthold, Ann-Beth Moller, Peter Azzopardi, Mariame Guèye Ba, Lucy Fagan, Valentina Baltag, Lale Say, Anshu Banerjee, Theresa Diaz

**Affiliations:** a Maternal, Newborn, Child and Adolescent Health Department, WHO, Geneva, Switzerland; b Reproductive Health and Research Department, WHO, Geneva, Switzerland; c Maternal and child health program, Burnet Institute, Melbourne, Australia; d Wardliparingga Aboriginal Research Unit, South Australian Health and Medical Research Institute, Adelaide, Australia; e Centre for Adolescent Health, Murdoch Children's Research Institute, University of Melbourne, Australia; f University Cheikh Anta Diop of Dakar, Faculty of Medicine, Pharmacy and Odontology/Gynecology and Obstetrics Clinic, University Teaching Hospital A. Le Dantec, Dakar, Senegal; g United Nations Major Group for Children and Youth, United Kingdom

Adolescents have shifted into the focus of global policy, reflecting their central role in achieving the 2030 Sustainable Development Goals (SDGs) [1], [2]. With 1.2 billion adolescents aged 10–19 years, the largest adolescent population in human history, representing more than 16% of the world's population, investments in adolescent health and well-being will yield benefits not only for adolescents now but also for their adult lives and future generations [3], [4].

The SDGs, the Global Strategy for Women's, Children's and Adolescent's Health, the Global Accelerated Action for the Health of Adolescents, and the Lancet Commission on Adolescent Health and Well-Being all emphasize the need for high-quality data to identify priorities and monitor progress in adolescent health [2], [4], [5], [6]. In response, there have been investments by United Nations (UN) agencies, country governments, and academia and other stakeholders around indicator development and data collection. These efforts have, however, largely occurred in silos with little harmonization across initiatives, resulting in duplication of work and inefficiencies, and yet a persistent lack of quality data required to inform effective, efficient, and accountable investments for adolescent health and well-being [7].

To harmonize measurement efforts and improve the quality and coverage of adolescent health data, World Health Organizations' Departments for Maternal, Newborn, Child and Adolescent Health and Reproductive Health and Research, in collaboration with the UN H6 partnership agencies, [2] have established an Advisory Group on “*Global Action for Measurement of Adolescent health–GAMA.*” The Global Action for Measurement of Adolescent Health (GAMA) Advisory Group was established alongside the *Child Health Accountability Tracking (CHAT)* Advisory Group, both following the example of the *Mother and Newborn Information for Tracking Outcomes and Results* (MoNITOR) Advisory Group, established in 2015 [8], [9]. Together, the GAMA, *CHAT*, and MoNITOR Advisory Groups aim to improve health measurement and reporting during key phases of the life course so as to ensure accountable action.

The GAMA Advisory Group consists of 12 experts, one MoNITOR representative, and four young professionals (Table 1), selected through a competitive process. Members were selected based on their technical expertise including adolescent health epidemiology, monitoring and evaluation, survey design, indicator development, and health information systems implementation. Further considerations in the selection were to ensure broad expertise across the main health issues of adolescents, geographic diversity, and gender balance. Members of the Advisory Group were appointed to serve for an initial term of 2 years.

**Table 1:** Global Action for Measurement of Adolescent health (GAMA) Advisory Group members

Name	Gender	Country of origin	Affiliation
Co-chairs			
Peter Azzopardi	M	Australia	Burnet Institute, Melbourne; Wardliparingga Aboriginal Research Unit, Adelaide; Centre for Adolescent Health, University of Melbourne
Mariame Guèye Ba	F	Sénégal	University Cheikh Anta Diop of Dakar, Faculty of Medicine, Pharmacy and Odontology/Gynecology and Obstetrics Clinic, University Teaching Hospital A. Le Dantec, Dakar
Lucy Fagan (young professional)	F	United Kingdom	UN Major Group for Children and Youth
Experts			
Emmanuel Adebayo	M	Nigeria	Federal University of Agriculture, Abeokuta
Krishna Bose	F	India	Bill & Melinda Gates Institute for Population and Reproductive Health, Johns Hopkins Bloomberg School of Public Health, Baltimore
Saeed Dastgiri	M	Iran	Tabriz Health Services Management Research Center, Tabriz University of Medical Sciences, Tabriz
Jane Ferguson	F	Canada/Switzerland	Independent Consultant on adolescent health and development
Ann Hagell	F	United Kingdom	Association for Young People's Health, London
Joanna Inchley	F	United Kingdom	MRC/CSO Social and Public Health Sciences Unit, University of Glasgow, Glasgow
Laura Kann	F	USA	Independent Consultant
Sunil Mehra	M	India	MAMTA Health Institute for Mother and Child, New Delhi
Elizabeth Saewyc	F	Canada	School of Nursing, University of British Columbia, Vancouver
Kun Tang	M	China	Research Center for Public Health, Tsinghua University Medical School, Beijing
Alison Morgan (representing MoNITOR)	F	Australia	Nossal Institute for Global Health, Melbourne School of Population and Global Health, University of Melbourne, Melbourne
Young professionals			
Charity Giyava	F	Zimbabwe	Women Deliver
Dakshitha Wickremarathne	M	Sri Lanka	Youth Advocacy Network, Sri Lanka
Diana Yeung	F	USA	Johns Hopkins Bloomberg School of Public Health, Baltimore

The GAMA Advisory Group had their first meeting from November 28–30, 2018, in Geneva, Switzerland, and considered the current landscape of adolescent health measurement, including key surveys such as the Demographic and Health Surveys [10], the Multiple Indicator Cluster Survey [11], the Health Behavior in School-Aged Children [12], the Global School-Based Student Health Survey [13], and the Global Early Adolescent Study [14], initiatives to improve mortality data for adolescents and measurement around mental health, nutrition, sexual and reproductive health, substance use, and violence. The group also reviewed existing indicators for adolescent health, facilitated by a mapping of key initiatives and indicator compilations [2], [4], [5], [15], [16], [17], [18], [19].

Several shortcomings with current approaches to adolescent health measurement were identified: First, indicators, their definitions and assessment methods across indicator frameworks are inconsistent, poorly harmonized with data availability, and incompletely aligned to needs. For example, adolescent overweight and obesity, a well-recognized population health risk for future and intergenerational health [20], was inconsistently measured across indicator frameworks and notably missing from the SDGs. Second, age- and sex-disaggregated data for adolescents are often lacking, making the use of the data for program planning difficult. Third, current measurement does not evenly address some adolescent subgroups, for example, out-of-school, in humanitarian settings, boys, migrants, young people of diverse gender identity, incarcerated adolescents, and ethnic and religious minorities. Fourth, topic areas with data gaps included mental health, injury, positive measures of adolescent health and well-being, and measures of sexual and reproductive health among unmarried adolescents and of sensitive nature where more qualitative research would be needed to move toward quantitative measures. Fifth, the link between global and national indicators, as well as between indicators and programming at national and subnational levels is often missing. Sixth, selection of indicators may be dictated by availability of data rather than the most important adolescent health issues. Seventh, data collection tools or administrative systems delivering data are often not well designed for adolescents. For example, registration of deaths drawn from health facilities will largely underestimate adolescent deaths, such as deaths from road traffic, that occur in communities.

The overarching goal of GAMA is to define a core set of adolescent health indicators by mid-2020 to converge data collection and reporting efforts. To achieve this, GAMA's initial work plan will concentrate around two key tasks carried out in collaboration with country partners and relevant organizations to ensure the work is relevant and recognized: The first is to define a conceptual framework with priority areas for adolescent health measurement globally, considering what needs to be measured from the perspectives of young people, policymakers, healthcare practitioners, and programmers, and taking into account the disease burden, opportunity for intervention, drivers of health inequality, and different contexts of various regions. Building on this, the second task will map existing indicators and available data of sufficient quality and coverage against the defined priorities. This process will help define indicators that are aligned with the priority areas but also identify data gaps. GAMA's work is documented on a Web site that will be available by May 2019 on WHO's home pages and will be further disseminated through publications and other reporting mechanisms of the involved UN agencies.

The GAMA Advisory Group acknowledges several challenges, including the tension of defining a core set of indicators while capturing the important differences that exist across the developmental continuum, the varying pattern of health needs and opportunity for response across different settings, and the important differences across genders. The task is large, but without agreement on core indicators, our efforts to address adolescent health are likely to be inefficient and ineffective, compromising the health of our young people, and all our futures.

## References

[1] Ki-moon B. (2016). Sustainability--engaging future generations now. Lancet.

[2] Every Woman Every Child (2015). The global strategy for women's, children's and adolescents' health (2016-2030).

[3] United Nations, Department of Economic and Social Affairs, Population Division (2017). Monitoring global population trends. http://www.un.org/en/development/desa/population/publications/index.shtml.

[4] Patton G.C., Sawyer S.M., Santelli J.S. (2016). Our future: A Lancet commission on adolescent health and wellbeing. Lancet.

[5] Inter-Agency and Expert Group on Sustainable Development Goal Indicators (2017). Revised list of global sustainable development goal indicators.

[6] World Health Organization (2017). Global accelerated action for the health of adolescents (AA-HA!): Guidance to support country implementation.

[7] Azzopardi P., Kennedy E., Patton G. (2017). Data and indicators to measure adolescent health, social development and well-being. Innocent Research Briefs 2017-04; Methods: conducting research with adolescents in low- and middle-income countries, No. 2.

[8] Moran A.C., Moller A.B., Chou D. (2018). ‘What gets measured gets managed’: Revisiting the indicators for maternal and newborn health programmes. Reprod Health.

[9] Moller A.B., Newby H., Hanson C. (2018). Measures matter: A scoping review of maternal and newborn indicators. PLoS One.

[10] United States Agency for International Development (2018). The DHS program. https://dhsprogram.com/.

[11] UNICEF (2018). Multiple Indicator Cluster Surveys. http://mics.unicef.org/.

[12] HBSC International Coordinating Centre, University of Glasgow (2018). Health behaviour in school-aged children. http://www.hbsc.org/.

[13] World Health Organization (2018). Global School-Based Student Health Survey (GSHS). https://www.who.int/ncds/surveillance/gshs/en/.

[14] World Health Organization (2018). Johns Hopkins Bloomberg School of Public Health, Global Early Adolescent Study. http://www.geastudy.org/.

[15] Countdown to 2030 (2018). Women's, children's and adolescents' health. http://countdown2030.org/.

[16] Family Planning 2020 (2018). FP 2020. https://www.familyplanning2020.org/.

[17] UNICEF (2018). Adolescent country tracker. https://data.unicef.org/resources/adolescent-country-tracker/.

[18] World Health Organization (2018). Global reference list of 100 core health indicators (plus health-related SDGs).

[19] World Health Organization (2015). Global reference list of health indicators for adolescents (aged 10-19 years).

[20] Patton G.C., Olsson C.A., Skirbekk V. (2018). Adolescence and the next generation. Nature.

